# Profiling the onset of somatic embryogenesis in *Arabidopsis*

**DOI:** 10.1186/s12864-017-4391-1

**Published:** 2017-12-29

**Authors:** E. Magnani, J. M. Jiménez-Gómez, L. Soubigou-Taconnat, L. Lepiniec, E. Fiume

**Affiliations:** 1 Insitut Jean-Pierre Bourgin (IJPB), INRA, AgroParisTech, CNRS, Université Paris-Saclay, INRA, Route de St-Cyr (RD10), 78026 Versailles Cedex, France; 2 POPS, Plateforme TranscriptOmique, Institute of Plant Sciences, Université Paris-Saclay, rue de Noetzlin, Plateau du Moulon, 91190 Gif-sur-Yvette, France

**Keywords:** Somatic embryogenesis, Totipotency, Callus cells, INTACT

## Abstract

**Background:**

Totipotency is the ability of a cell to regenerate a whole organism. Plant somatic embryogenesis (SE) is a remarkable example of totipotency because somatic cells reverse differentiation, respond to an appropriate stimulus and initiate embryo development. Although SE is an ideal system to investigate de-differentiation and differentiation, we still lack a deep molecular understanding of the phenomenon due to experimental restraints.

**Results:**

We applied the INTACT method to specifically isolate the nuclei of those cells undergoing SE among the majority of non-embryogenic cells that make up a callus. We compared the transcriptome of embryogenic cells to the one of proliferating callus cells. Our analyses revealed that embryogenic cells are transcriptionally rather than metabolically active. Embryogenic cells shut off biochemical pathways involved in carbohydrate and lipid metabolism and activate the transcriptional machinery. Furthermore, we show how early in SE, ground tissue and leaf primordia specification are switched on before the specification of a shoot apical meristem.

**Conclusions:**

This is the first attempt to specifically profile embryogenic cells among the different cell types that constitute plant in vitro tissue cultures. Our comparative analyses provide insights in the gene networks regulating SE and open new research avenues in the field of plant regeneration.

**Electronic supplementary material:**

The online version of this article (doi: 10.1186/s12864-017-4391-1) contains supplementary material, which is available to authorized users.

## Background

Plant developmental plasticity derives from its remarkable capacity of continuous growth, building upon a basic body plan that has been established during embryogenesis [1]. Thus, unlike animals [2], plants are equipped with pluripotent stem cells during their entire life span. In mammals, pluripotent stem cells have been successfully induced through manipulating the transcriptional and epigenetic networks of various differentiated cell types [3]. However, the factors that confer totipotency, the ability to give rise to cells in both embryonic and extra-embryonic lineages, are still elusive. Moreover, although it is currently unknown whether totipotency in metazoans can be induced and maintained in vitro [4], differentiated plant somatic cells can be induced in vitro to give rise to fully totipotent cells which develop into somatic embryos [5]. Many plant species, including *Arabidopsis thaliana* (*Arabidopsis*), respond well to somatic embryogenesis (SE) induction [6, 7]. This process is generally divided into two main steps: an induction phase and a developmental phase. During the induction phase, isolated somatic cells are subjected to conditions that promote cell proliferation and de-differentiation, and are believed to acquire the competence to undergo SE; during the developmental phase some of the cultured cells, under the right stimuli, start differentiating in somatic embryos [8]. A plethora of factors have been implicated in SE induction, including cell-to-cell signaling [9], cell wall composition alteration [10, 11], hormonal changes [12, 13] and epigenetic shifts [14, 15] but, in plants as well as in animals, the nature of totipotency has not yet been fully elucidated. In plant biology, the main limiting experimental factor has been the inability to specifically isolate and analyze those cells that are responding to SE induction, due to the lack of early cytological or morphological markers for SE. As a consequence, up to date, we do not precisely know the molecular mechanisms that cause some cells in the callus to change their fate and become embryogenic. In plants, establishment of purely embryogenic cultures has not yet been achieved, so we have to rely on primary cell isolation techniques from cultures where embryogenic cells are only a small percentage.

In this study, we analysed early molecular mechanisms that cause undifferentiated plant cells to become embryogenic through transcriptional characterization of embryogenic cells isolate with the INTACT technique from *Arabidopsis* in vitro cultures. The INTACT method has been developed to obtain reliable gene expression and chromatin profiling from specific cell-types [16, 17]. We found that embryogenic cells repress metabolic pathways and become more transcriptionally active. Moreover, we globally compare the transcriptome of both proliferating callus cells and embryogenic cells to other relevant tissues, thus gaining a general view on the nature of these cell-types.

## Results

### Isolation of nuclei from early embryogenic cells

**Fig. 1:**
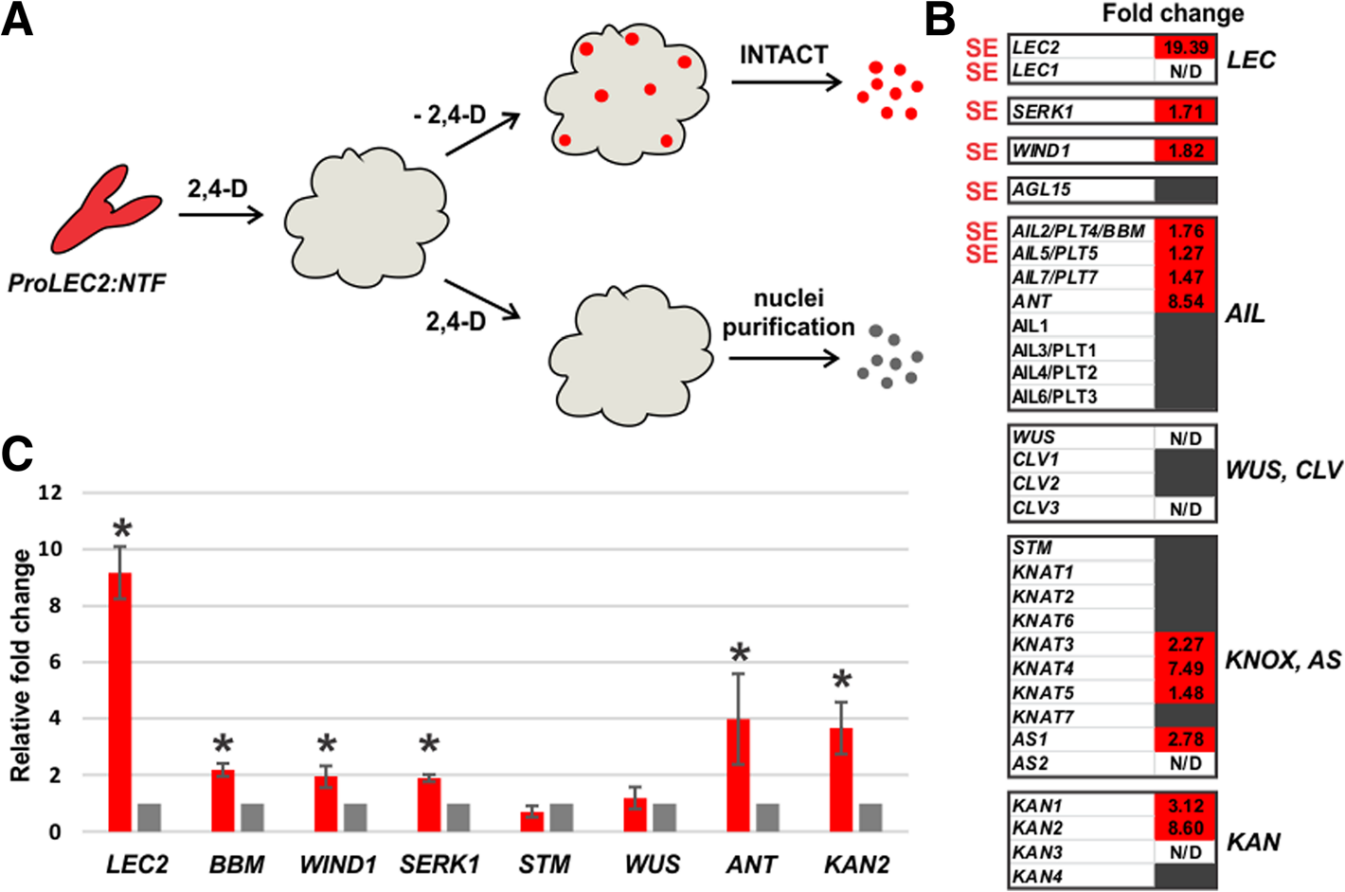
Embryogenic versus un-induced callus cells. **a** Experimental flow. *proLEC2:NTF* immature zygotic embryos were cultured on 2,4-D medium to induce callus formation. In vitro callus cultures were maintained on 2,4-D medium or transferred onto 2,4-D-free medium. Finally, nuclei were purified using the INTACT method or a standard nuclei purification method. Nuclei expressing the *NTF* tag are highlighted in red. **b** Fold change values in gene expression between embryogenic and un-induced callus cells as detected in DEGs analysis. Red and grey boxes indicate up-regulation in embryogenic cells and no statistical difference, respectively. N/D, not detected. Markers of somatic embryogenesis are marked “SE” in red. **c** Quantitative RT-PCR analyses of selected genes in embryogenic (red) and un-induced (grey) calli. Expression levels were normalized to *TUBULIN4* expression and were averaged from three independent biological samples. Values of un-induced callus samples are arbitrarily set to 1. Black asterisks indicate statistical difference between samples (Student’s *t* test, *P* < 0.01)

To gain new insights in the molecular processes that cause some cells in the callus to undergo somatic embryogenesis (SE), we produced INTACT-suitable *Arabidopsis* transgenic plants carrying the NTF chimeric protein under the control of the *LEAFY COTYLEDON 2* (*LEC2*) promoter region. The NTF protein consists of three domains: the WPP domain of *Arabidopsis* RAN GTPASE ACTIVATING PROTEIN 1, which is necessary and sufficient for nuclear envelope association, the green fluorescent protein (GFP) for visualization, and the biotin ligase recognition peptide (BLRP), which acts as a substrate for the *Escherichia coli* biotin ligase BirA (constitutively expressed in the INTACT transgenic lines background) [16]. LEC2 is a B3 domain transcription factor essential for proper development of the zygotic embryo and for triggering somatic embryogenesis in vegetative cells in the absence of exogenous auxin or stress treatments [18]. *LEC2* expression in embryogenic cultures has been documented in many plant species [19–21], making it a first-choice marker for SE. Two parallel in vitro embryogenic callus cultures were initiated from *ProLEC2*:*NTF* transgenic lines (in a *Pro35S:BirA* background). As expected, the NTF protein was visible in the immature zygotic embryos used to establish the culture (Additional file 1: Fig. S1A). As early as 3 days after callus induction on medium supplemented with 2,4-D, GFP fluorescence could not be detected and stayed off throughout callus formation and proliferation (Additional file 1: Figure S1B-E). After 3 weeks, one of the cultures was kept proliferating on 2,4-D, whereas the other was moved onto hormone-free medium to induce SE. After 10 days of SE induction, we detected GFP expression although no embryo structure was apparent on the callus surface (Additional file 1: Figure S1F and G). At this point, nuclei from embryogenic cultures induced to undergo SE and expressing the NTF marker were purified with the INTACT method whereas un-induced embryogenic callus cultures were subjected to nuclei purification (Fig. 1a). Following the INTACT pull-down, we exclusively retrieved nuclei bound to streptavidin-coated beads (Additional file 1: Figure S2A-C), most of which were associated in large clumps (Additional file 1: Figure S2A, red arrowheads). On the other hand, in control experiments carried with *ProLEC2*:*NTF* plants in a wild-type background, free nuclei were observed before INTACT pull-down (Additional file 1: Figure S2D, green arrowheads), and only beads were retrieved after pull-down (Additional file 1: Figure S2E, yellow arrowhead). Nuclear RNAs from two independent biological replicates of each sample were extracted and deep sequenced. We calculated expression values for each gene in each replicate and controlled the similarity of the samples using the log likelihood ratio statistic under a simple Poisson model [22]. As expected, replicates from each cell type clustered together and separated well from the replicates of the other cell type, revealing different expression profiles between the two cell types (Additional file 1: Figure S3). In total, we detected 17,576 genes in the two cell types. Remarkably, 98.8% of expressed genes were detected in embryogenic and proliferating callus cells, suggesting that we sampled early enough to look at the first differentiation events. Among the 1,2% of genes only expressed in one cell type, only 79 were uniquely detected in callus cells, and 134 were uniquely detected in embryogenic cells (Additional file 2: Table S1). To study differences in gene expression programs between the two cell types, we identified differentially-expressed genes (DEGs). A union set of 6699 DEGs (Additional file 3: Table S2), equal to 38% of detected genes, were observed with a balanced distribution between genes up and down-regulated in embryogenic (3327 genes were upregulated in callus cells and 3372 in embryogenic cells), suggesting that, at this stage, distinct cell fates are dictated by differential expression levels rather than cell specific gene expression. In order to validate our transcriptome data, we checked the transcription level of endogenous *LEC2* and other genes known to play a role in SE and differentiation (Fig. 1b). *LEC2* features among the most dramatically upregulated genes, as expected from our experimental set-up. Nevertheless, LEC2 transcript abundance is relatively low compared to other DEGs (Fig. 1b and Additional file 3: Table S2). This is in accordance to previous transcriptome analyses performed on *Arabidopsis* embryogenic cultures [23], indicating that low levels of LEC2 transcription factor are sufficient to trigger the developmental cascade that leads to SE. However, transcripts of *LEC1*, another marker for SE [24], were not detected. *LEC1* expression is positively regulated by *LEC2* [25], suggesting that we might have sampled our cultures before *LEC1* transcriptional activation by *LEC2*. Consistent with this, another transcription factor controlled by *LEC2*, the MADS domain AGAMOUS-LIKE15 (AGL15) [26], was also not upregulated in embryogenic cells.

Beyond LEC transcription factors, a handful of genes have been implicated in the transition from somatic to embryonic fate in plants. Similarly to what has been observed with LEC1 and LEC2, over-expression of the AINTEGUMENTA*-*LIKE (AIL) BABYBOOM (BBM) and AIL5 transcription factors promote embryogenesis and organogenesis in the absence of exogenously applied growth regulators [27, 28], whereas the *SOMATIC EMBRYOGENESIS RECEPTOR KINASE1* (*SERK1*) has been shown to enhance somatic embryo development [9]. We found upregulation of *BBM*, *AIL5* and *SERK1* in embryogenic cells, though to a much lesser extent than *LEC2* (Fig. 1b). Interestingly, their absolute expression level is high in both callus cells and embryogenic cells (Additional file 3: Table S2), suggesting that they might play a different role than *LEC2*, and might be involved in the acquisition of competence to undergo SE (induction phase), rather than triggering embryo differentiation (developmental phase). Consistent with this interpretation, *BBM* was recently found to directly and positively regulate *LEC2* and *LEC1* expression [29]. The AP2/ERF transcription factor WOUND INDUCED DEDIFFERENTIATION 1 (WIND1) has been implicated in establishing and maintaining the de-differentiation status of somatic cells upon wounding, and seedlings over-expressing *WIND1* exhibit callus-like un-organized cell proliferation around the shoot meristem [30]. In our experimental system, *WIND1* exhibits the same pattern observed for *BBM* and *SERK1* (Fig. 1b), suggesting that it might play a similar role in conferring embryogenic competence. Finally, qRT-PCR analyses on embryogenic callus and un-induced callus confirmed expression trends for all genes tested (Fig. 1c). Detection of relatively early markers of SE and the strong enrichment in *LEC2* transcripts indicate that we have correctly purified SE-induced cells, whereas the absence of *LEC2* induced markers, suggests that we have sampled cultures at an early stage of SE.

Last, to verify that our culture conditions were suitable for producing embryogenic callus, we left part of our callus material on hormone-free medium for up to 3 weeks in order to observe somatic embryos emergence. Additional file 1: Fig. S4 shows somatic embryos emerging from embryogenic callus (Additional file 1: Figure S4A) and an optical section of a developing somatic embryo (Additional file 1: Figure S4B) obtained through confocal microscopy.

### Early embryogenic cells are transcriptionally rather than metabolically active

**Fig. 2:**
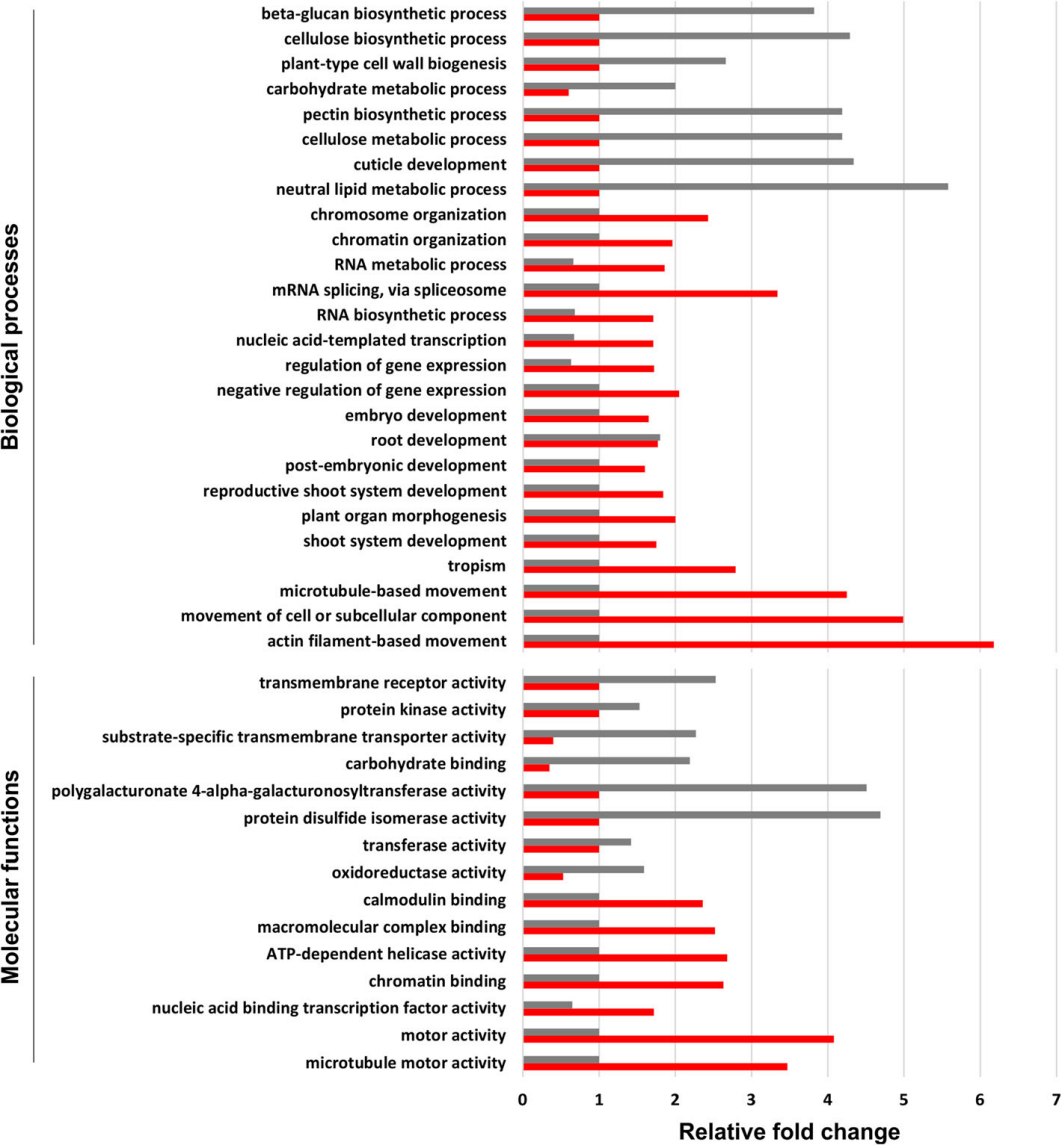
GO enrichment analysis of DEGs. Relative enrichment of GO terms in “molecular functions” and “biological processes” categories for differentially-expressed genes up-regulated in embryogenic (red) and un-induced (grey) callus cells. Only statistically different categories are shown. Unchanged values in either sample were arbitrarily set to one

To study the differences between embryogenic and non-induced callus cell types from a functional and molecular point of view, we performed gene ontology studies for DEGs. Our analyses show that DEGs up-regulated (DEGsUP) and DEGs down-regulated (DEGsDOWN) in embryogenic cells fall into different gene ontology categories (Additional file 3: Table S2 and Fig. 2), implying that different transcriptional programs are active in callus and embryogenic cells. In the ‘Biological process’ category, over-represented GO terms for DEGsUP include ‘actin filament-based movement’, ‘movement of cell or subcellular component’ and ‘microtubule-based movement’ (Fig. 2), suggesting a re-organization of cell contents in embryogenic cells, possibly due to changes in cell fate and activation of polarized cell growth. Other GO terms over-represented in DEGsUP include ‘chromosome organization’, and ‘chromatin organization’ (Fig. 2), in line with mounting evidences that epigenetic marks act as gatekeepers to cell fate transitions [31, 32]. Notably, the ‘regulation of gene expression’ category is over-represented in DEGsUP and under-represented in DEGsDOWN. We quantified the number of differentially expressed transcription factors (TFs) and found that they account for 14.5% of DEGsUP and for only 4.2% (64 on 1539) of DEGsDOWN (64 out of 1539, percentages are statistically different *p* < 0.01, Fisher Exact test). This suggests that a boost of expression of transcription factors is likely to cause the activation of SE developmental pathways, or the repression of callus fate. On the other hand, among the DEGsDOWN, we observed an enrichment of gene categories linked to a variety of metabolic activities such as ‘neutral lipid metabolic process’, ‘cellulose biosynthetic process’ and ‘plant-type cell wall biogenesis’. These results have been further confirmed by gene enrichments studies in the ‘molecular function’ category (Fig. 2): over-represented GO terms for DEGsUP include ‘chromatin binding’, ‘nucleic acid binding transcription factor activity’ and ‘motor activity’, whereas over-represented GO terms for DEGsDOWN feature terms related to biochemical activities such as ‘polygalacturonate 4-alpha-galacturonosyltransferase activity’, ‘carbohydrate binding’, ‘substrate-specific transmembrane transporter activity’ and ‘neutral lipid metabolic processes’ (Fig. 2). Overall, these analyses suggested that embryogenic cells fate is associated to an enhanced transcriptional activity and repression of metabolic pathways.

### Early embryogenic cells share similarities with meristematic and embryo cells at the transcriptional level

**Fig. 3:**
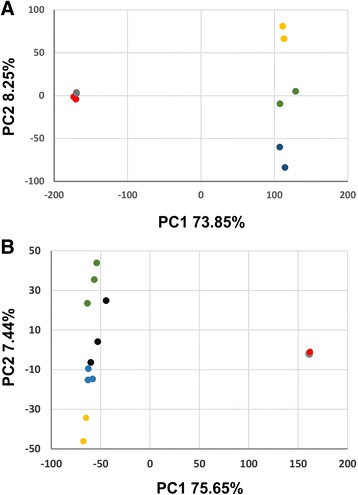
Embryogenic and callus cells resemble octant stage embryos and the peripheral domain of the SAM. Embryogenic cells and callus cells are shown in red and gray respectively. Each dot represents an experimental replicate. **a** PCA comparing callus and embryogenic cells to early stages of embryo development. **S**eeds carrying 1–2 cell embryos, 8 cell embryos (octant), and 32 cell embryos (globular stage) samples are shown in yellow, green, and blue, respectively. **b** PCA comparing callus and embryogenic cells to various sub-domains of the SAM. Stem cell niche marked by *CLV3* expression, SAM without *CLV3* expressing domain, SAM organizing center marked by *WUS* expression, and SAM peripheral zone marked by *FILAMENTOUS FLOWER* (*FIL*) expression samples are highlighted in green, black, yellow and blue, respectively

**Fig. 4:**
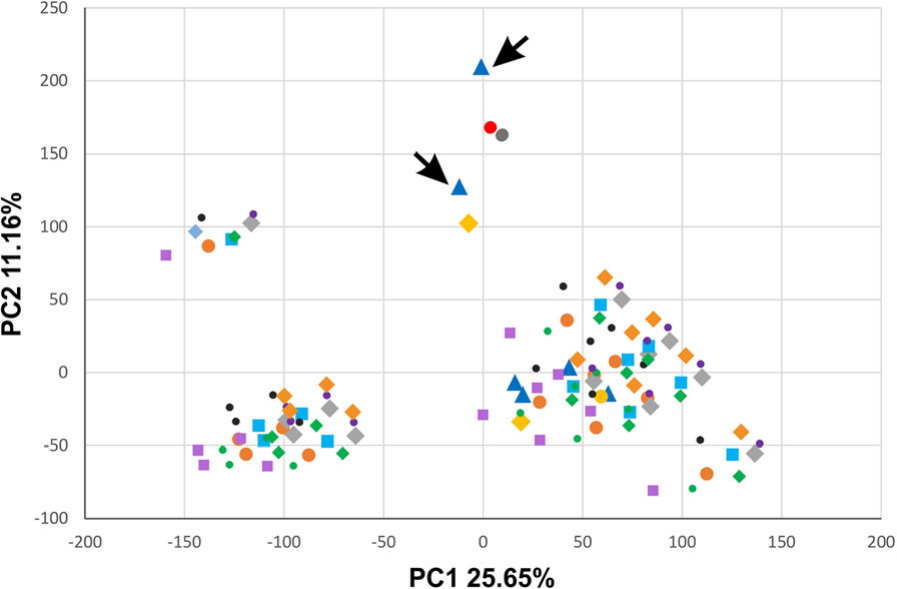
Embryogenic and callus cells resemble the LRC domains adjacent to the root tip and the columella cells. PCA comparing callus and embryogenic cells to various root cell types at different developmental stages. Embryogenic cells and callus cells are shown in red and gray respectively. Blue triangles: LRC (transverse sections 1 to 6), the two LRC samples closest to the root tip are pointed by arrows. Yellow diamonds: columella cells. Gray diamonds: endodermis (transverse sections 2 to 13). Green diamonds: phloem pole (transverse sections 2 to 13). Orange dots: cortex (transverse sections 2 to 13). Cyan squares: Root hair (transverse sections 2 to 13). Black dots: non-hair cells (transverse sections 2 to 13). Purple squares: xylem pole (transverse sections 2 to 13). Purple dots: phloem (transverse sections 2 to 13). Orange diamonds: metaprotophloem (transverse sections 2 to 13). Yellow dot: quiescent center. Each dot represents an experimental replicate for embryogenic and callus cells, and a different transverse section along the root’s longitudinal axis (according to Brady et al., 2007)

We studied the transcriptional set up of embryogenic cells their resemblance to other cell types. For this, we performed principal component analysis (PCA) including both collected data sets (embryogenic cells and proliferating callus cells) together with publicly available expression data (microarrays or RNA deep sequencing collected from different *Arabidopsis* tissues). In all PCA analyses, embryogenic cells and callus cells clustered close to each other (Figs. 3 and 4). Although, to a great extent, this is expected when comparing data from different experiments, the tight clustering of our samples might be the result of early sampling during somatic embryogenesis and suggests that we are looking at the first differentiation steps between these two cell types. This interpretation is in line with the finding of a large gene expression overlap between the two samples. Somatic embryos have been widely reported to resemble both morphologically and physiologically zygotic embryos [8, 33, 34]. In order to assess similarities between our cultures and developing zygotic embryos, we performed a PCA using our datasets together with publicly available sequencing data from 1 to 2 cell embryos, 8 cell embryos (octant) and 32 cell embryos (globular stage) [35]. Whereas the first principal component separates samples by experiment, the second component accounts for more than 8% of the variance in the dataset and correlates well with embryo development. According to PC2, embryogenic cells are closely related to 8-cell embryos (Fig. 3a, green dots) and well differentiated from 2 or 32-cell embryos (Fig. 3a, yellow and blue dots respectively). In accordance to this result, we detected low or no transcripts for *WUSCHEL* (*WUS*), *SHOOTMERISTEMLESS* (*STM*) and *CLAVATA3* (*CLV3*), meristematic genes whose expression has been documented in embryogenic culture systems (Fig. 1b and Additional file 4: Table S3) [12, 36–38]. Indeed, during zygotic embryo development, markers of an organized shoot apical meristem (SAM) are visible only starting at the 16 cell stage, with *WUS* expression appearing in four sub-epidermal apical cells [39], later followed by *STM* activation in the apical domain of the early globular embryo [40] and last, *CLV3* expression is detected at the heart stage between the emerging cotyledons [41]. On the other hand, we did not detect *WUSCHEL-related-HOMEOBOX 2* (*WOX2*) and *WOX9*, known to establish the apical and the basal domains of the early zygotic embryo [42]. Lack of *WOX2* and *WOX9* expression suggests that early patterning during somatic embryos establishment might be directed by alternative developmental routes. Other members of the family belonging to the *WOX2* module (*WOX1,2,3,5*) recently reported to initiate the stem cell program during zygotic embryogenesis [43] were not detected, exception made for *WOX5*, which nonetheless it is not differentially expressed in embryogenic cells. The HD-ZIP III genes *PHABULOSA* (*PHB*), *PHAVOLUTA* (*PHV*), and *REVOLUTA* (*REV*) are well known factors playing a fundamental role in establishing the SAM in zygotic embryos. They are expressed throughout the 16-cells stage embryo, and in later stages their expression is restricted to the central region of the embryo (SAM included), provasculature and the cotyledons adaxial side [44, 45]. We found these genes highly expressed in both callus and embryogenic cells, together with other *HD-ZIP III* genes: *CORONA* (*CNA*) and *Arabidopsis thaliana HOMEOBOX GENE8* (*ATHB8*). *REV*, *CNA* and *ATHB8* are listed among the DEGsUP, arguing that SAM specification during SE takes alternative developmental routes to those known to function during zygotic embryogenesis.

Given the documented expression of SAM markers in a variety of embryogenic cultures [12, 36, 38], it is generally believed that SAM organization is one of the early events in SE. Thus, we performed a PCA analysis to compare the expression patterns of callus cells and embryogenic cells to those from SAM functional subdomains [46]. Namely, we used expression profiles from the stem cell niche marked by *CLV3* expression, the organizing center marked by *WUS* expression and the SAM peripheral zone marked by *FILAMENTOUS FLOWER* (*FIL*) expression. As shown above, the first principal component explained 75.6% of the variance and differentiated well our dataset from the SAM dataset. However, when principal components 2 was considered (explaining 7.4% of the variance), we observed a closer similarity of the embryonic cells to the *pFIL* expressing domain of the SAM rather than the stem-cell niche or the *WUS* expressing organizing center (Fig. 3b). This is in line with the lack of transcript for the well-known regulators of stem cell activity *WUS*, *CLV3*, *CLV1* and *CLV2* [39, 47] (Fig. 1b). Despite we did not detect *FIL* expression or other members of the *YABBY* family in embryogenic cells (Additional file 3: Table S2 and Additional file 4: Table S3), *YABBY* positive regulators *KANADI 1* (*KAN1*) and *KAN2* [48, 49] are among the DEGsUP (Fig. 1b and Additional file 3: Table S2). Furthermore, we observed low or no transcript levels of *class I KNOTTED-like homeobox* (*KNOX1*) genes *STM*, *KNOTTED1-LIKE HOMEOBOX GENE 1* (*KNAT1*), *KNAT2* and *KNAT6* [50]. By contrast, three out of four members of the *class II KNOX* (*KNOX2*) genes, *KNAT3*, *KNAT4*, and *KNAT5*, are among our DEGsUP (Fig. 1b-c and Additional file 3: Table S2; Additional file 4: Table S3). *KNOX1* and *KNOX2* have been shown to have antagonistic and opposing functions, with *KNOX1* involved in maintenance of meristematic potential in the SAM and *KNOX2* implicated in leaf primordia formation [51]. The Myb transcription factor ASYMMETRIC LEAVES 1 (AS1), whose expression is detected in young leaf primordia, acts in antagonism with KNOX1 transcription factors [52], and is up-regulated in embryogenic cells in our study (Fig. 1b and Additional file 3: Table S2; Additional file 4: Table S3).

Members of the AIL transcription factors are known to play a central role in embryogenesis, meristem maintenance, organ positioning and growth [53]. Together with upregulation of the two *AIL* members (*AIL5* and *BBM*) known to play a role in SE (discussed above), we observed upregulation of *ANT* and *AIL7* (Fig. 1b-c and Additional file 4: Table S3). Similarly to what is observed with the *KNOX* gene family, among the *AIL* genes, we observed upregulation of members involved in the development of the meristem peripheral zone and young leaf primordia [53, 54], whereas other members implicated in maintenance of the stem cell niche (such as *AIL3*, *AIL4*, and *AIL6*) [53, 54], were not found differentially expressed (Fig. 1b and Additional file 4: Table S3), suggesting that SAM peripheral zone markers might switch on before stem cell niche ones during SE.

In 2010, Sugimoto et al. have shown how callus induced by the application of auxin and cytokinin to in vitro cultured *Arabidopsis* tissues, is characterized by gene expression patterns reminiscent of root meristems, even if it is derived from aerial organs [55]. We performed PCA using our samples and publicly available microarray expression profiles of a high-resolution set of developmental time points within a single *Arabidopsis* root [56]. The first principal component explained 25.57% of the variance and differentiated root tissues and our datasets, whereas the second (explaining the 11.16% of the variance) suggested a closer similarity of our datasets to meristematic tissues of the root tip, such as the proximal lateral root cap (LRC) (Fig. 4, blue triangles pointed by arrows) rather than other root tissues, or more distal parts of the LRC (Fig. 4). This result is in line with the observations previously made by Sugimoto et al. [55], linking callus cell fate to meristematic root tissue fate. Moreover, this PCA is supported by expression of well-known markers for endodermis and LRC specification in our samples (Additional file 4: Table S3). The NAC domain transcription factors FEZ, SOMBRERO (SMB) and BEARSKIN1 (BRN1) are important factors in patterning the root tip by controlling cell division planes and root cap maturation [57, 58] and their transcripts are more abundant in callus cells rather than embryogenic cells, as they feature in our DEGsDOWN list (Additional file 3: Table S2 and Additional file 4: Table S3). On the other hand, ground tissues specific markers *SCARECROW* (*SCR*), *SHORTROOT* (*SHR*), *JACKDAW* (*JKD*) and *CYCLIN D6* (*CYCD6*) are all upregulated in embryogenic cells (Additional file 3: Table S2 and Additional file 4: Table S3). *SCR*, together with *SHR* is known to play a role in both root and shoot endodermis specification in *Arabidopsis* [59], thus, their expression in embryogenic cells is a sign of ongoing tissue specification and patterning.

## Discussion

In this study, we have compared the transcriptome of non-embryogenic and embryogenic callus cells in early stages of somatic embryo development (Fig. 1a). We sampled embryogenic culture before any embryo morphological structure became apparent on callus surface, with the aim of taking an early transcriptional snapshot of cell differentiation towards SE. Expression profiling of the two cell populations and informatics analyses revealed gene expression networks specific to embryogenic cells and proliferating callus cells (Additional file 2: Table S1). Gene ontology studies on DEGs showed how embryogenic cells are transcriptionally active, likely undergoing subcellular re-organization, and activation of chromatin modifications. On the other hand, they showed repression of biochemical pathways linked to carbohydrate and lipid metabolism (Fig. 2).

We observed enrichment of known markers of SE in embryogenic cells (Fig. 1b and c) including *LEC2*, whose regulatory sequence was used to build our SE marker for INTACT. These results confirm that we have enriched our samples in embryogenic cells. Two *LEC2* regulated markers of SE were either not detected (*LEC1*) or did not show any change in transcripts levels (*AGL15*), suggesting that we sampled cultures before LEC2 transcription factor levels reached a critical point for activation of these two targets. Future experiments designed to collect samples at different time points during SE induction will shed some light on the timing of *LEC1* and *AGL15* induction.

Expression of the stem cell marker *WUS* in embryogenic cultures has been widely documented [12, 60]. In our experimental set up, however, *WUS*, together with *CLV3*, *STM*, *CLV1* and *CLV2* were either not detected or did not show upregulation in embryogenic cells (Fig. 1b and c). We interpret this data as a sign of sampling before the organization of a functional SAM. In accordance with this interpretation, PCA analysis comparing the transcriptome of embryogenic cells to the transcriptome of embryos at early developmental stages (Fig. 3a), revealed a stronger similarity with seeds bearing octant stage zygotic embryos (Fig. 3a, green dots) rather than 1–2 cell stage or globular stage embryos (Fig. 3a, yellow and blue dots respectively). At this stage of zygotic embryo development, the stem cell marker genes are not yet expressed [39, 41, 61], corroborating the interpretation that we have sampled our cultures at early stages of somatic embryo development, before the specification of a SAM. Lack of *WUS* expression at early stages of SE is not totally surprising in the light of new studies that show its dispensability for stem cell initiation during zygotic embryogenesis [43]. Furthermore, although gain-of-function studies suggest that *WUS* is involved in the promotion and/or maintenance of totipotent cells [62], *Arabidopsis wus* mutant is still able to undergo somatic, suggesting that multiple pathways can lead to totipotency. On the other hand, *WOX2,* together with its paralogs *WOX1*, *WOX3*, and *WOX5* were found to play a fundamental role in stem cell fate initiation during zygotic embryogenesis by positively regulating the HD-ZIP III transcription factors PHV, PHB and REV [43]. Exception made for *WOX5*, which was found expressed in both embryogenic and callus cells, *WOX1*, *WOX2*, and *WOX3* were not detected in our samples (Additional file 4: Table S3). These results suggest that we either sampled before stem cell fate specification through the expression of the *WOX1235,* or that SAM specification is achieved by alternative developmental pathways. Most intriguingly, we detected high levels of the HD-ZIP III transcription factors PHB, PHV, REV and CNA in both callus cells and embryogenic cells, with only CNA and REV upregulated in embryogenic cells (Additional file 4: Table S3), showing the dispensability of the *WOX1235* module to induce HD-ZIP III transcription factors in callus cultures. Nevertheless, at this point, we cannot exclude that transient *WOX1235* expression in un-induced callus induced *HD-ZIP III* expression. According to our observations, SAM specification during SE is achieved by developmental mechanisms that act downstream of the HD-ZIP III transcription factors. *Arabidopsis WOX5* is a marker of the root quiescent center and root primordia initiation [63–65]. More recently, rice WOX5 has been identified as a marker of callus cell formation [66], indicating a wider role in tissue de-differentiation and cell fate transitions.

Lack of stem cell niche markers further correlates with PCA analysis comparing embryogenic cells to different subdomains of the SAM (Fig. 3b). The expression profile of embryogenic cells better correlates with cells belonging to the *FIL* domain of the SAM peripheral zone, rather than with the *CLV3* or *WUS* expressing subdomains. This finding implies that cells undergoing SE up-regulate genes involved in organ initiation (such as *ANT*, *AS1*, *KNOX2* genes and *KAN2*) before inducing genes associated with SAM stem cell or niche fate (Additional file 4: Table S3). On the other hand, PCA analyses found embryogenic cells transcriptionally similar to octant stage zygotic embryos, a stage that still lacks bilateral symmetry both morphologically and molecularly. We interpret these findings as a sign of developmental divergence between SE and zygotic embryogenesis at early stages. Although fully developed somatic and zygotic embryos are similar at the morphological and molecular level, they arise in totally different conditions and might then originate from totally different morphogenic pathways that converge to a certain degree at some point in development. In an alternative scenario, the lack of some expected markers for SE could be due to our choice of *LEC2* as a SE marker. First of all, we cannot exclude that upon SE induction, different cells responding to the induction signal might express different subsets of genes, making the embryogenic callus a transcriptionally heterogeneous tissue. Along this line of thought, the stochastic spatial arrangement of different cell types, rather than a single embryogenic cell type, might give rise to somatic embryo development. Second, the INTACT approach requires the use of upstream regulatory regions alone, as the nuclear envelope targeting domain WPP needs to be placed at the amino-terminus of the chimeric NTF protein. This technical requirement could have excluded potential regulatory sequences found either in *LEC2* introns or coding sequences. Although we have used a *LEC2* regulatory sequence shown to recapitulate *LEC2* expression in developing zygotic embryos and to fully complement *lec2–4* loss of function allele [67], we cannot exclude that additional genomic sequence might be important for *LEC2* regulation during SE. These possibilities will be addressed in the future, by performing parallel experiments utilizing a variety of markers for SE.

Furthermore, in the past, callus has been widely believed to be an un-differentiated cellular state [68, 69]. Nonetheless, callus includes cells with various degrees of differentiation [68] and more recently, transcriptional profiling of *Arabidopsis* callus derived from different tissues has shown great similarity to root meristem cells [55]. Comparing the transcriptome profiles of early embryogenic and callus cells to cells derived from different root tissues, we observed a closer similarity to the proximal part of the LRC (Fig. 4, blue triangles). This result was further supported by the expression analysis of relevant gene families involved in root tissue specification. Markers of the epidermis and LRC *FEZ*, *SMB* and *BRN1*, are all expressed in our cell types. As expected, they are upregulated in callus cells, in accordance with the view of callus cells acquiring a fate close to a root tip cell fate. Thus, during SE, their expression needs to be repressed to allow the action of other patterning genes, such as the shoot and root ground tissues patterning genes *SCR*, *SHR*, *JKD* and *CYCD6*, all found upregulated in embryogenic cells.

## Conclusions

Overall, this study produced a first early transcriptional snapshot of selected cells undergoing SE and expressing the *LEC2* marker using the INTACT method. Our analyses revealed that embryogenic cells diverge from callus cell fate by generally repressing biochemical pathways and root meristem genes while switching on transcriptional networks involved in shoot patterning, cellular re-organization and polarized cell growth. These findings open new and interesting avenues to study both the onset of SE and the de novo organization of meristematic tissues.

## Methods

### Plant material

Transgenic lines were produced transforming *Arabidopsis thaliana* transgenic plants (ecotype Columbia) constitutively expressing the *Escherichia coli* biotin ligase BirA. *Agrobacterium tumefaciens* strain GV3101 was used to transform plants by the floral dip method [70]. Transgenic plants were selected on Murashige and Skoog medium supplemented with appropriate selecting agents.

### In vitro tissue culture

Immature zygotic embryos at the torpedo stage of development were manually isolated from sterilized siliques and cultured on a Gamborg B5 basal salt medium (Duchefa, 3.16 g/l) supplemented with sucrose (20 g/l), MES (0.5 g/l), Phytagel (5 g/l) and 2,4-D (7uM). Explants were kept in the dark at 16 °C for 3 weeks to promote callus growth. To induce somatic embryogenesis, part of the callus cultures was moved on 2,4-D free medium and kept in the same conditions until sampling.

### INTACT

Before proceeding to nuclei purification through the INTACT method, samples have been frozen in liquid nitrogen. INTACT experiments and nuclei isolation have been conducted as previously described by Deal and Henikoff [16].

#### INTACT vector

We modified the gateway plasmid *pMDC107* [71], replacing the *mgfp6* sequence with the *NTF* sequence, in order to obtain an INTACT vector (*pMDC107-NTF*) where we could easily clone the promoter region of interest by gateway technology (Invitrogen). We partially digested *pMDC107* with the EcoRI restriction enzyme to cut the vector at position 1. Subsequently, we cut with AscI to remove the sequence containing the *mgfp6* and the *nos terminator*. The cut plasmid was then subjected to ends fill-in with the T4 DNA polymerase. A blunt PCR amplified sequence containing the *NTF* sequence and the *nos terminator* (from the *ADF8:NTF* plasmid kindly provided by the Henikoff laboratory [16]), was ligated into the modified vector and later transformed into the ccdB survival cells (Invitrogen). Colonies were screened to select vectors with the insert in the wanted orientation. Plasmid DNA from positive colonies has been sequenced to ensure the cloning sites were not rearranged.

### RNA extraction

RNA was isolated using the *mir*Vana miRNA isolation kit (Ambion), following the manufacturer’s instructions for total RNA preparations.

### RT-PCR, qRT-PCR

First-strand cDNA synthesis was performed on 700 μg of total RNA using Superscript III RNase H- reverse transcriptase (Invitrogen), according to the manufacturer’s instructions. The annealing temperature was 56 to 58 °C for all primer pairs. Quantification of transcripts by real-time qPCR was performed using SsoAdvanced Universal SYBR Green Supermix (Bio-Rad) and CFX Connect Real-Time PCR Detection System (Bio-Rad). Three technical replicates were run for each sample. The specificity of the amplification was determined by performing a dissociation curve analysis. Relative quantification values were calculated using the 2-ΔCt method [72].

### RNA deep sequencing and data processing

RNA-seq libraries were prepared using the SMARTer Stranded RNA-Seq Kits (Takara). RNA-seq experiments were carried with an IG-CNS Illumina Hiseq2000. The RNA-seq samples have been sequenced in paired-end (PE) with a sizing of 260 bp and a read length of 100 bases. Four samples by lane of Hiseq2000 using individual bar-coded adapters and giving approximately 30 million of PE reads by sample were generated. To facilitate comparisons, each RNA-Seq sample followed the same pipeline from trimming to count of transcript abundance as follows. Read preprocessing criteria included trimming library adapters and performing quality control checks using FastQC. The raw data (fastq) were trimmed by fastx toolkit for Phred Quality Score > 20, read length > 30 bases, and the ribosome sequences were removed with tool sortMeRNA [73]. The mapper Bowtie 2 [74] was used to align reads against the *Arabidopsis* gene database TAIR10 (http://www.arabidopsis.org/) with one isoform per gene corresponding to the representative gene model. The abundance of each gene was calculated by a local script which parses SAM files and counts only reads that map unambiguously one gene, removing multi-hits. According to these rules, around 90% of PE reads were associated to a gene, 3 to 4% could not be mapped and 4 to 5% resulted in multi-hits and were discarded. For the differential analysis, genes which did not have at least 1 read after a count per million (CPM) normalization in at least one half of the samples were discarded. Library size was normalized using the method TMM and count distribution was modeled with a negative binomial generalized linear model where the harvest date was taken into account. Dispersion was estimated by the edgeR method (Version 1.12.0) [75] in the statistical software ‘R’ (Version 2.15.0, R Development Core Team). Expression differences were compared using likelihood ratio test and *p* values were adjusted by the Benjamini-Hochberg procedure to control FDR. A gene was declared differentially expressed if its adjusted p value was lower than 0.05.

### Gene ontology studies

Gene ontology enrichment analyses were conducted at the Gene Ontology Consortium (http://geneontology.org/) website, using the “Enrichment analysis tool” and the annotation data sets “PANTHER GO-slim Biological Processes” and “PANTHER GO-slim Molecular Processes”. Only results with *P* < 0.05 are discussed in this paper.

### PCA analyses

PCA analysis comparing the transcriptome of embryogenic cells and embryos at early developmental stages was performed by merging the raw read counts publicly available in Nodine MD and Bartel DP [35] (GEO series: GSE33713) with the raw read counts from our RNA-seq experiments. The merged raw read counts were normalized using the rlogTransformation function in DESeq2 package [76]. PCA analysis was performed on the resulting normalized values using the prcomp function in R.

We also performed PCA analysis comparing the expression patterns in our RNA-seq samples to that of cells derived from different root tissues obtained from the BAR website (http://bar.utoronto.ca). Expression values from the Root Tissue series were downloaded from BAR (http://bar.utoronto.ca/ntools/cgi-bin/ATGE_Root_raw.txt), natural log transformed and merged with the rlog normalized expression values from our RNA-seq data obtained with DESeq2. IN order to equalize the distribution of expression values between the microarray and RNA-seq datasets, we removed from the analyses low expressed genes in our RNA-seq dataset as indicated by the Cook’s distance cutoff in the DESeq2 package. This filtering resulted in a matrix of 16,532 genes that was used for PCA analysis with the prcomp function in R.

### Pseudo-Schiff propidium iodide mPS-PI staining and microscopy

This protocol allows the staining of cell walls of fixed plant material as developed by Xu et al., 2016 [77]. mPS-PI imaging was conducted with a Leica TCS-SP5 spectral 304 confocal laser scanning microscope (Leica Microsystems). GFP and DAPI fluorescence was detected using an epi-fluorescence (Zeiss Axiozoom).

## Additional files

Additional file 1: Figure S1. ProLEC2:NTF line. (A) GFP fluorescence image of a ProLEC2:NTF zygotic embryo. Bar = 50 μm. The inset shows a cotyledon close-up (green channel: GFP, red channel: chlorophyll). (B-C) Transmitted light (B) and GFP fluorescence image (C) of an isolated ProLEC2:NTF zygotic embryo on 2,4-D. Bar = 100 μm. (D-E) Transmitted light (D) and GFP (E) fluorescence image of a ProLEC2:NTF callus on 2,4-D. Bar = 500 μm. (F-G) Transmitted light (F) and GFP (G) fluorescence image of a ProLEC2:NTF callus on 2,4-D free medium. Bar = 500 μm. **Figure S2.** Purification of nuclei from embryogenic callus using INTACT. (A) DAPI fluorescence image of beads and ProLEC2:NTF nuclei (in Pro35S:BirA background) isolated from embryogenic callus. Bar = 50 μm. (B-C) DAPI (B) and GFP (C) fluorescence image of a ProLEC2:NTF (in Pro35S:BirA background) nucleus surrounded by beads isolated from embryogenic callus. Bar = 10 μm. (D-E) DAPI fluorescence image of ProLEC2:NTF nuclei (in wild-type background) and beads before (D) and after (E) INTACT. Bar = 50 μm. Red, yellow and green arrowheads indicate nuclei-beads clumps, isolated beads, and isolated nuclei, respectively. **Figure S3.** Similarity in expression patterns between samples in the experiment. Read counts per gene were used to calculate the Poisson dissimilarity matrix between samples as implemented in the PoiClaClu package in R. Differences in color represent differences in expression profiles between samples and are represented in a heatmap. **Figure S4.** Somatic embryos. (A) Multiple somatic embryos emerging from embryogenic callus. Scale bar = 500 μm. (B) Optical longitudinal section of a somatic embryo (mPS-PI imaging technique). Scale bar = 50 μm. (DOCX 3601 kb)

Additional file 2: Table S1. Genes uniquely expressed in embryogenic cells (sheet 1) and callus cells (sheet2). Genes uniquely expressed in proliferating callus. (XLSX 29 kb)

Additional file 3: Table S2. Differentially expressed genes between embryogenic cells and callus cells. (XLSX 942 kb)

Additional file 4: Table S3. Expression data concerning all the genes discussed in the main text. (XLSX 16 kb)

## Abbreviations

DEGs: Differentially expressed genes
INTACT: Isolation of nuclei tagged in specific cell types
SE: Somatic Embryogenesis

## References

[1] Jeong S (2016). Going mainstream: how is the body axis of plants first initiated in the embryo?. Dev Biol.

[2] Condic ML (2014). Totipotency: what it is and what it is not. Stem Cells Dev.

[3] Smith ZD, Sindhu C, Meissner A (2016). Molecular features of cellular reprogramming and development. Nat Rev Mol Cell Biol.

[4] Lu F, Zhang Y (2015). Cell totipotency: molecular features, induction, and maintenance. Natl Sci Rev.

[5] Smertenko A, Bozhkov PV (2014). Somatic embryogenesis: life and death processes during apical-basal patterning. J Exp Bot.

[6] Gaj MD (2011). Somatic embryogenesis and plant regeneration in the culture of Arabidopsis Thaliana (L.) Heynh. Immature zygotic embryos. Methods Mol Biol.

[7] Pillon E (1996). A protocol for obtaining embryogenic cell lines from Arabidopsis. Plant J.

[8] Luo Y, Koop HU (1997). Somatic embryogenesis in cultured immature zygotic embryos and leaf protoplasts of Arabidopsis Thaliana ecotypes. Planta.

[9] Hecht V (2001). The Arabidopsis SOMATIC EMBRYOGENESIS RECEPTOR KINASE 1 gene is expressed in developing ovules and embryos and enhances embryogenic competence in culture. Plant Physiol.

[10] Majewska-Sawka A, Nothnagel EA (2000). The multiple roles of arabinogalactan proteins in plant development. Plant Physiol.

[11] Malinowski R, Filipecki M (2002). The role of cell wall in plant embryogenesis. Cell Mol Biol Lett.

[12] Su YH (2009). Auxin-induced WUS expression is essential for embryonic stem cell renewal during somatic embryogenesis in Arabidopsis. Plant J.

[13] Zheng Q (2016). Gene regulation by the AGL15 transcription factor reveals hormone interactions in somatic embryogenesis. Plant Physiol.

[14] De-la-Pena C (2015). The role of chromatin modifications in somatic embryogenesis in plants. Front Plant Sci.

[15] Mozgova I, Munoz-Viana R, Hennig L (2017). PRC2 represses hormone-induced somatic embryogenesis in vegetative tissue of Arabidopsis Thaliana. PLoS Genet.

[16] Deal RB, Henikoff S (2011). The INTACT method for cell type-specific gene expression and chromatin profiling in Arabidopsis Thaliana. Nat Protoc.

[17] Moreno-Romero J (2017). Applying the INTACT method to purify endosperm nuclei and to generate parental-specific epigenome profiles. Nat Protoc.

[18] Stone SL (2001). LEAFY COTYLEDON2 encodes a B3 domain transcription factor that induces embryo development. Proc Natl Acad Sci U S A.

[19] Uddenberg D (2011). Embryogenic potential and expression of embryogenesis-related genes in conifers are affected by treatment with a histone deacetylase inhibitor. Planta.

[20] Zhang Y (2014). The Theobroma Cacao B3 domain transcription factor TcLEC2 plays a duel role in control of embryo development and maturation. BMC Plant Biol.

[21] Guo F (2013). Induced expression of AtLEC1 and AtLEC2 differentially promotes somatic embryogenesis in transgenic tobacco plants. PLoS One.

[22] Witten DM (2011). Classification and clustering of sequencing data using a Poisson model. Ann Appl Stat.

[23] Wickramasuriya AM, Dunwell JM (2015). Global scale transcriptome analysis of Arabidopsis embryogenesis in vitro. BMC Genomics.

[24] Lotan T (1998). Arabidopsis LEAFY COTYLEDON1 is sufficient to induce embryo development in vegetative cells. Cell.

[25] Stone SL (2008). Arabidopsis LEAFY COTYLEDON2 induces maturation traits and auxin activity: implications for somatic embryogenesis. Proc Natl Acad Sci U S A.

[26] Braybrook SA (2006). Genes directly regulated by LEAFY COTYLEDON2 provide insight into the control of embryo maturation and somatic embryogenesis. Proc Natl Acad Sci U S A.

[27] Boutilier K (2002). Ectopic expression of BABY BOOM triggers a conversion from vegetative to embryonic growth. Plant Cell.

[28] Tsuwamoto R, Yokoi S, Takahata Y (2010). Arabidopsis EMBRYOMAKER encoding an AP2 domain transcription factor plays a key role in developmental change from vegetative to embryonic phase. Plant Mol Biol.

[29] Horstman A (2017). The BABY BOOM transcription factor activates the LEC1-ABI3-FUS3-LEC2 network to induce somatic embryogenesis. Plant Physiol.

[30] Iwase A (2011). The AP2/ERF transcription factor WIND1 controls cell dedifferentiation in Arabidopsis. Curr Biol.

[31] Ikeuchi M (2015). PRC2 represses dedifferentiation of mature somatic cells in Arabidopsis. Nat Plants.

[32] Shemer O (2015). Competency for shoot regeneration from Arabidopsis root explants is regulated by DNA methylation. Plant Sci.

[33] Willemsen V, Scheres B (2004). Mechanisms of pattern formation in plant embryogenesis. Annu Rev Genet.

[34] Jin F (2014). Comparative transcriptome analysis between somatic embryos (SEs) and zygotic embryos in cotton: evidence for stress response functions in SE development. Plant Biotechnol J.

[35] Nodine MD, Bartel DP (2012). Maternal and paternal genomes contribute equally to the transcriptome of early plant embryos. Nature.

[36] Elhiti M (2010). Modulation of embryo-forming capacity in culture through the expression of Brassica genes involved in the regulation of the shoot apical meristem. J Exp Bot.

[37] Elhiti M, Stasolla C (2011). Ectopic expression of the Brassica SHOOTMERISTEMLESS attenuates the deleterious effects of the auxin transport inhibitor TIBA on somatic embryo number and morphology. Plant Sci.

[38] Bouchabke-Coussa O (2013). Wuschel overexpression promotes somatic embryogenesis and induces organogenesis in cotton (Gossypium Hirsutum L.) tissues cultured in vitro. Plant Cell Rep.

[39] Mayer KF (1998). Role of WUSCHEL in regulating stem cell fate in the Arabidopsis shoot meristem. Cell.

[40] Long JA, Barton MK (1998). The development of apical embryonic pattern in Arabidopsis. Development.

[41] Tucker MR (2008). Vascular signalling mediated by ZWILLE potentiates WUSCHEL function during shoot meristem stem cell development in the Arabidopsis embryo. Development.

[42] Haecker A (2004). Expression dynamics of WOX genes mark cell fate decisions during early embryonic patterning in Arabidopsis Thaliana. Development.

[43] Zhang Z (2017). A molecular framework for the embryonic initiation of shoot Meristem stem cells. Dev Cell.

[44] Emery JF (2003). Radial patterning of Arabidopsis shoots by class III HD-ZIP and KANADI genes. Curr Biol.

[45] McConnell JR (2001). Role of PHABULOSA and PHAVOLUTA in determining radial patterning in shoots. Nature.

[46] Yadav RK (2009). Gene expression map of the Arabidopsis shoot apical meristem stem cell niche. Proc Natl Acad Sci U S A.

[47] Fletcher JC (1999). Signaling of cell fate decisions by CLAVATA3 in Arabidopsis shoot meristems. Science.

[48] Eshed Y (2004). Asymmetric leaf development and blade expansion in Arabidopsis are mediated by KANADI and YABBY activities. Development.

[49] Eshed Y (2001). Establishment of polarity in lateral organs of plants. Curr Biol.

[50] Hay A, Tsiantis M (2009). A KNOX family TALE. Curr Opin Plant Biol.

[51] Furumizu C (2015). Antagonistic roles for KNOX1 and KNOX2 genes in patterning the land plant body plan following an ancient gene duplication. PLoS Genet.

[52] Rast MI, Simon R (2012). Arabidopsis JAGGED LATERAL ORGANS acts with ASYMMETRIC LEAVES2 to coordinate KNOX and PIN expression in shoot and root meristems. Plant Cell.

[53] Horstman A (2014). AINTEGUMENTA-LIKE proteins: hubs in a plethora of networks. Trends Plant Sci.

[54] Mudunkothge JS, Krizek BA (2012). Three Arabidopsis AIL/PLT genes act in combination to regulate shoot apical meristem function. Plant J.

[55] Sugimoto K, Jiao Y, Meyerowitz EM (2010). Arabidopsis regeneration from multiple tissues occurs via a root development pathway. Dev Cell.

[56] Brady SM (2007). A high-resolution root spatiotemporal map reveals dominant expression patterns. Science.

[57] Willemsen V (2008). The NAC domain transcription factors FEZ and SOMBRERO control the orientation of cell division plane in Arabidopsis root stem cells. Dev Cell.

[58] Bennett T (2010). SOMBRERO, BEARSKIN1, and BEARSKIN2 regulate root cap maturation in Arabidopsis. Plant Cell.

[59] Yoon EK (2016). Conservation and diversification of the SHR-SCR-SCL23 regulatory network in the development of the functional endodermis in Arabidopsis shoots. Mol Plant.

[60] Zheng W (2014). AtWuschel promotes formation of the embryogenic callus in Gossypium Hirsutum. PLoS One.

[61] Barton MK (2010). Twenty years on: the inner workings of the shoot apical meristem, a developmental dynamo. Dev Biol.

[62] Zuo J (2002). The WUSCHEL gene promotes vegetative-to-embryonic transition in Arabidopsis. Plant J.

[63] Sarkar AK (2007). Conserved factors regulate signalling in Arabidopsis Thaliana shoot and root stem cell organizers. Nature.

[64] Petricka JJ, Benfey PN (2011). Reconstructing regulatory network transitions. Trends Cell Biol.

[65] Kong X (2015). WOX5 is shining in the root stem cell niche. Trends Plant Sci.

[66] Hu B (2017). Divergent regeneration-competent cells adopt a common mechanism for callus initiation in angiosperms. Regeneration.

[67] Berger N (2011). Transcriptional regulation of Arabidopsis LEAFY COTYLEDON2 involves RLE, a cis-element that regulates trimethylation of histone H3 at lysine-27. Plant Cell.

[68] Ikeuchi M, Sugimoto K, Iwase A (2013). Plant callus: mechanisms of induction and repression. Plant Cell.

[69] Braun AC (1959). A demonstration of the recovery of the crown-gall tumor cell with the use of complex tumors of single-cell origin. Proc Natl Acad Sci U S A.

[70] Clough SJ, Bent AF (1998). Floral dip: a simplified method for Agrobacterium-mediated transformation of Arabidopsis Thaliana. Plant J.

[71] Curtis MD, Grossniklaus U (2003). A gateway cloning vector set for high-throughput functional analysis of genes in planta. Plant Physiol.

[72] Livak KJ, Schmittgen TD (2001). Analysis of relative gene expression data using real-time quantitative PCR and the 2(−Delta Delta C(T)) method. Methods.

[73] Kopylova E, Noe L, Touzet H (2012). SortMeRNA: fast and accurate filtering of ribosomal RNAs in metatranscriptomic data. Bioinformatics.

[74] Langmead B, Salzberg SL (2012). Fast gapped-read alignment with bowtie 2. Nat Methods.

[75] McCarthy DJ, Chen Y, Smyth GK (2012). Differential expression analysis of multifactor RNA-Seq experiments with respect to biological variation. Nucleic Acids Res.

[76] Love MI, Huber W, Anders S (2014). Moderated estimation of fold change and dispersion for RNA-seq data with DESeq2. Genome Biol.

[77] Xu W (2016). The antagonistic development of endosperm and nucellus in Arabidopsis seeds. Plant Cell.

