# Supplementary material for: Profiling the onset of somatic embryogenesis in Arabidopsis
Source: BMC Genomics. 2017 Dec 29;18:998. doi: 10.1186/s12864-017-4391-1 (PMC5747089; doi:10.1186/s12864-017-4391-1)
Supplement: Supplementary file 1 — ProLEC2:NTF line. (A) GFP fluorescence image of a ProLEC2:NTF zygotic embryo. Bar = 50 μm. The inset shows a cotyledon close-up (green channel: GFP, red channel: chlorophyll). (B-C) Transmitted light (B) and GFP fluorescence image (C) of an isolated ProLEC2:NTF zygotic embryo on 2,4-D. Bar = 100 μm. (D-E) Transmitted light (D) and GFP (E) fluorescence image of a ProLEC2:NTF callus on 2,4-D. Bar = 500 μm. (F-G) Transmitted light (F) and GFP (G) fluorescence image of a ProLEC2:NTF callus on 2,4-D free medium. Bar = 500 μm. Figure S2. Purification of nuclei from embryogenic callus using INTACT. (A) DAPI fluorescence image of beads and ProLEC2:NTF nuclei (in Pro35S:BirA background) isolated from embryogenic callus. Bar = 50 μm. (B-C) DAPI (B) and GFP (C) fluorescence image of a ProLEC2:NTF (in Pro35S:BirA background) nucleus surrounded by beads isolated from embryogenic callus. Bar = 10 μm. (D-E) DAPI fluorescence image of ProLEC2:NTF nuclei (in wild-type background) and beads before (D) and after (E) INTACT. Bar = 50 μm. Red, yellow and green arrowheads indicate nuclei-beads clumps, isolated beads, and isolated nuclei, respectively. Figure S3. Similarity in expression patterns between samples in the experiment. Read counts per gene were used to calculate the Poisson dissimilarity matrix between samples as implemented in the PoiClaClu package in R. Differences in color represent differences in expression profiles between samples and are represented in a heatmap. Figure S4. Somatic embryos. (A) Multiple somatic embryos emerging from embryogenic callus. Scale bar = 500 μm. (B) Optical longitudinal section of a somatic embryo (mPS-PI imaging technique). Scale bar = 50 μm. (DOCX 3601 kb) [file 12864_2017_4391_MOESM1_ESM.docx]

Supplemental Figures

**
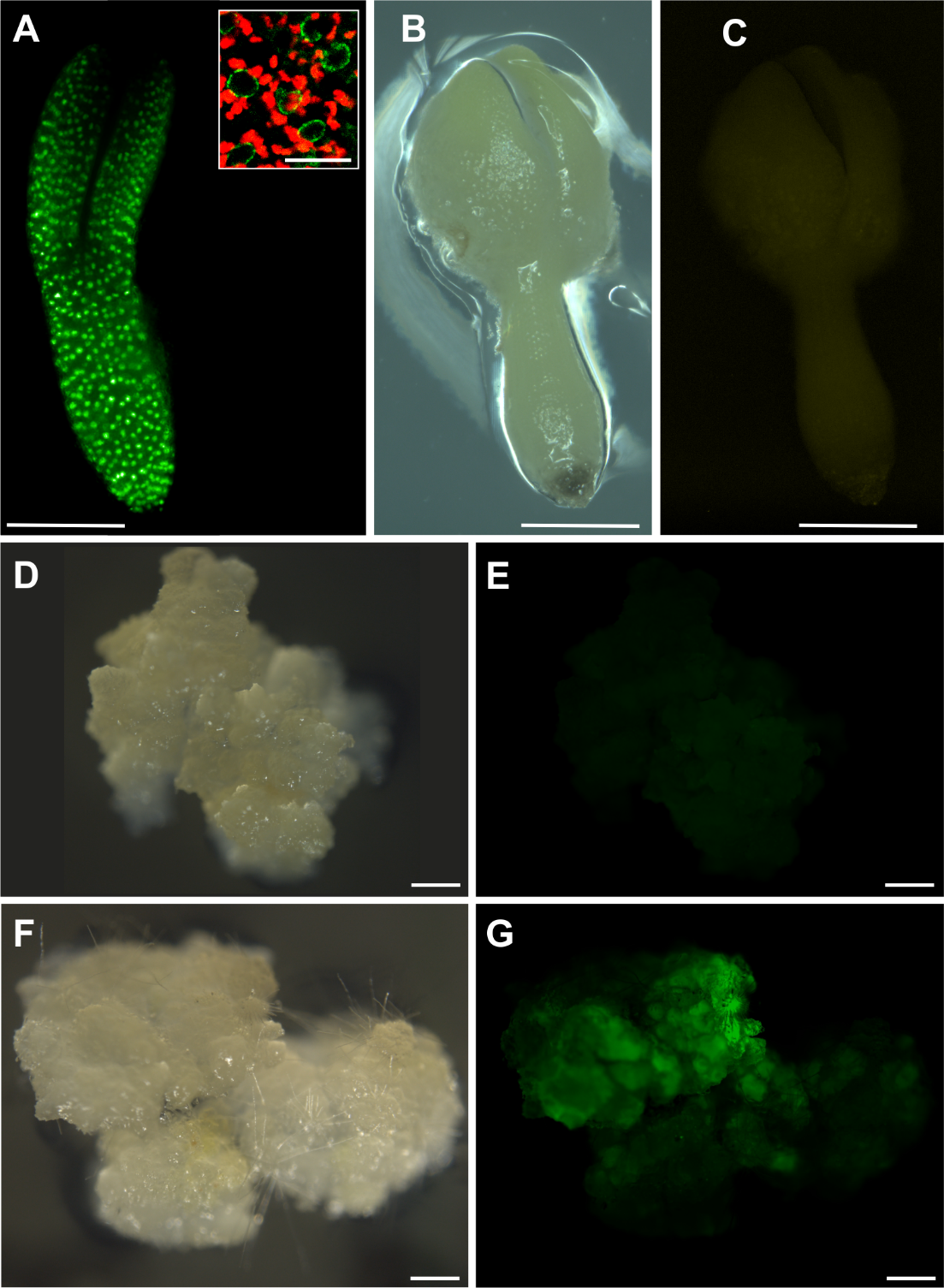
**

**Figure S1 INTACT *ProLEC2:NTF* line**

**(A)** GFP fluorescence image (epifluorescence microscope) of a *ProLEC2:NTF* immature zygotic embryo. Bar = 50 µm. The insert shows a GFP (green channel) and chlorophyll (red channel) fluorescence image (confocal laser microscope) of *ProLEC2:NTF* cotyledon cells.

**(B-C)** Transmitted light (B) and GFP fluorescence image (C) of an isolated *ProLEC2:NTF* immature zygotic embryo on 2,4-D medium. Bar = 100 µm.

**(D-E)** Transmitted light (D) and GFP (E) fluorescence image (epifluorescence microscope) of a *ProLEC2:NTF* callus on 2,4-D medium. Bar = 500 µm.

**(F-G)** Transmitted light (F) and GFP (G) fluorescence image (epifluorescence microscope) of a *ProLEC2:NTF* callus on 2,4-D free medium. Bar = 500 µm.


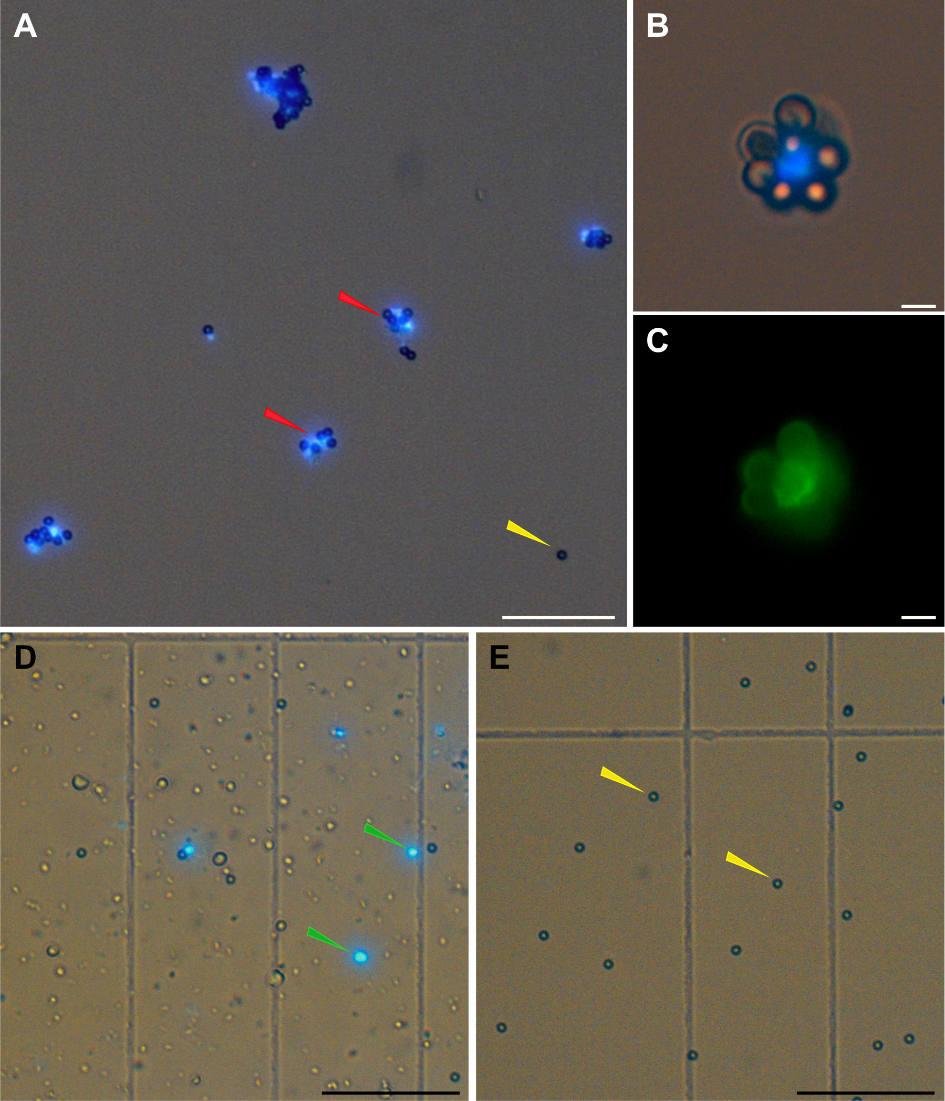


**Figure S2 Purification of nuclei from embryogenic callus cells using the INTACT method**

**(A)** DAPI fluorescence image (epifluorescence microscope) of beads and *ProLEC2:NTF* nuclei (in *Pro35S:BirA* transgenic background) isolated from embryogenic calli using the INTACT method. Bar = 50 µm.

**(B-C)** DAPI (B) and GFP (C) fluorescence image (epifluorescence microscope) of a *ProLEC2:NTF* (in *Pro35S:BirA* transgenic background) nucleus surrounded by beads isolated from embryogenic calli using the INTACT method. Bar = 10 µm

**(D-E)** DAPI fluorescence image (epifluorescence microscope) of *ProLEC2:NTF* nuclei (in wild-type background) and beads before (D) and after (E) INTACT pull-down. Bar = 50 µm.

Red, yellow and green arrowheads indicate nuclei-beads clumps, isolated beads, and isolated nuclei, respectively.


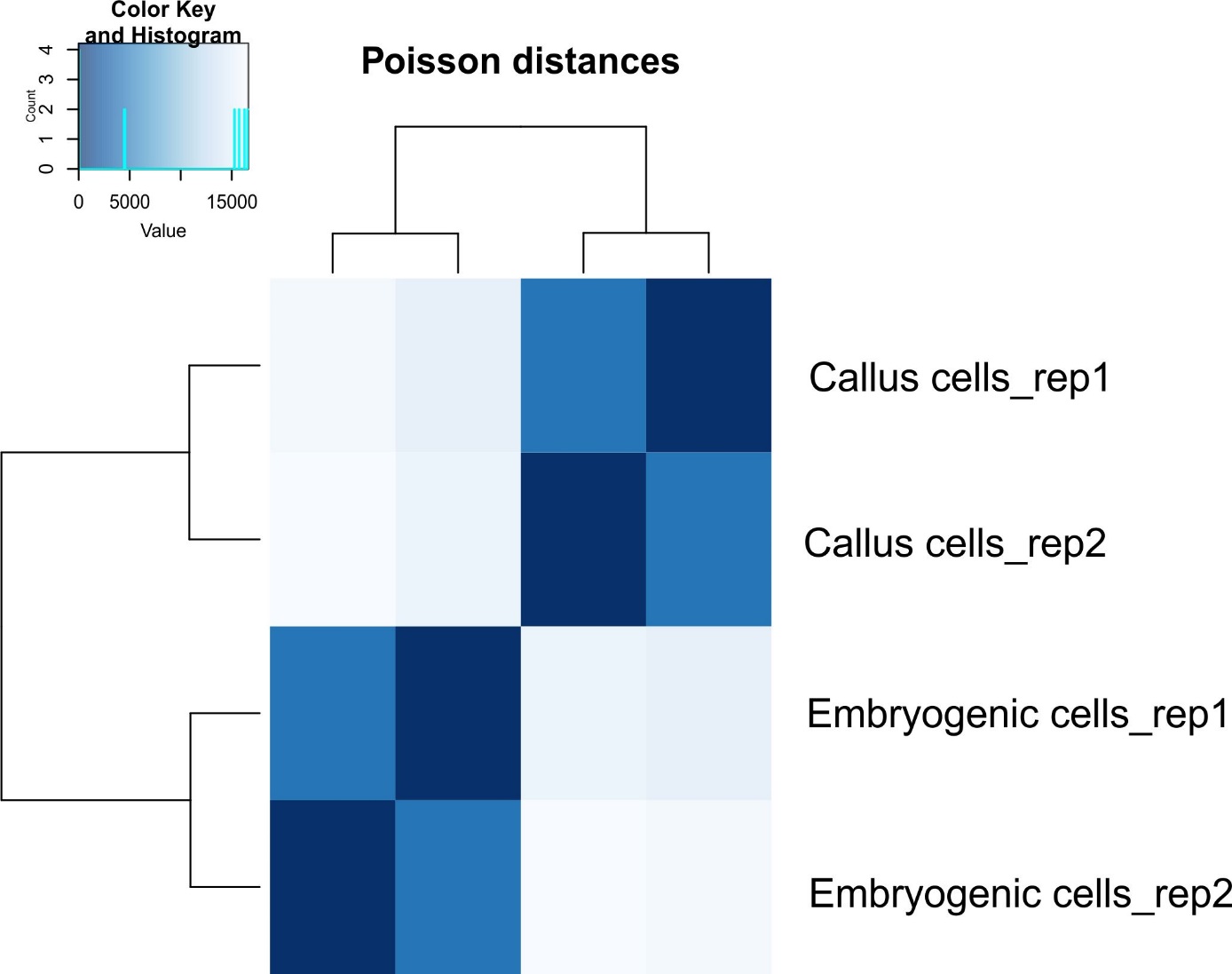


**Figure S3 Similarity in expression patterns between samples in the experiment.**

Read counts per gene were used to calculate the Poisson dissimilarity matrix between samples as implemented in the PoiClaClu package in R. Differences in color represent differences in expression profiles between samples and are represented in a heatmap.


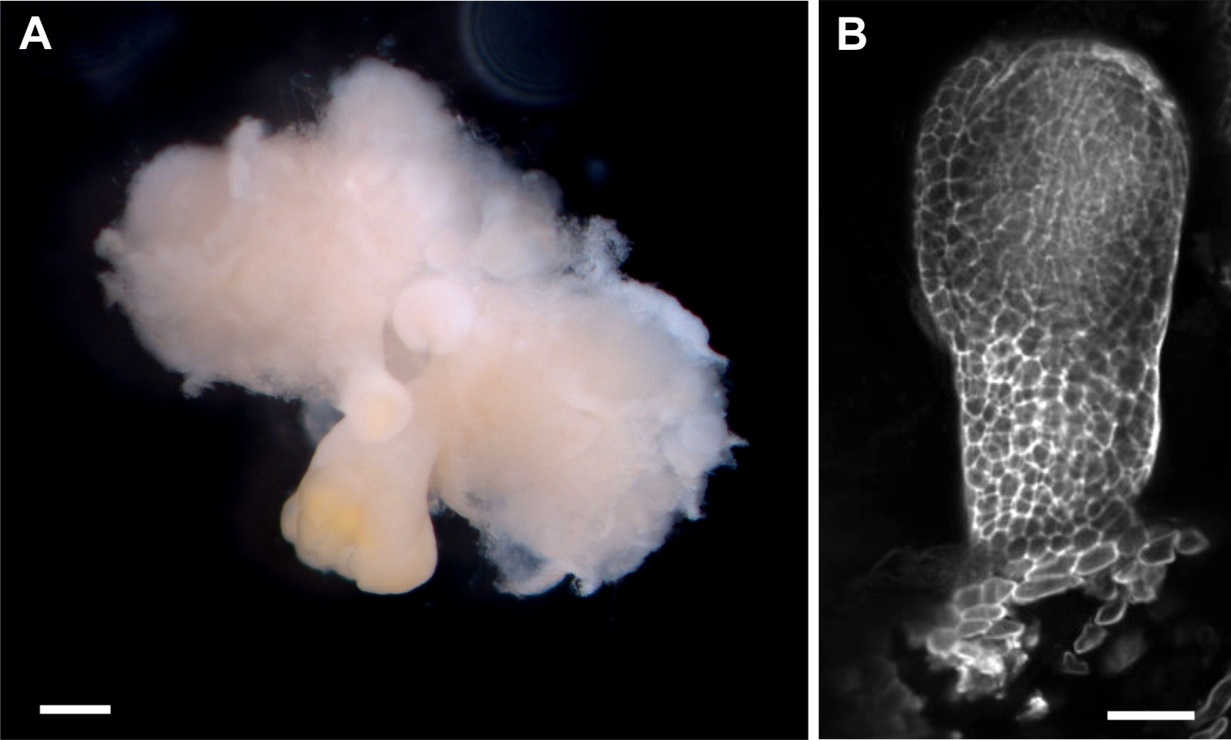


**Figure S4 Somatic embryos emerging from embryogenic callus.**

**(A)** Multiple somatic embryos emerging from embryogenic callus. Scale bar= 500 µm. **(B)** Optical longitudinal section of a somatic embryo imaged using the mPS-PI technique. Scale bar= 50 µm.
